# Risk Stratification in Acute Coronary Syndromes: The Systemic Immune-Inflammation Index as Prognostic Marker

**DOI:** 10.3390/medsci13030116

**Published:** 2025-08-08

**Authors:** Elena Emilia Babes, Andrei-Flavius Radu, Noemi Adaus Cretu, Gabriela Bungau, Camelia Cristina Diaconu, Delia Mirela Tit, Victor Vlad Babes

**Affiliations:** 1 Doctoral School of Biomedical Sciences, University of Oradea, 410087 Oradea, Romania; eebabes@uoradea.ro (E.E.B.); gbungau@uoradea.ro (G.B.); dtit@uoradea.ro (D.M.T.); 2 Department of Medical Disciplines, Faculty of Medicine and Pharmacy, University of Oradea, 410073 Oradea, Romania; vvbabes@uoradea.ro; 3 Department of Psycho-Neurosciences and Recovery, Faculty of Medicine and Pharmacy, University of Oradea, 410073 Oradea, Romania; 4 Faculty of Medicine, University of Medicine and Pharmacy Carol Davila Bucharest, 050474 Bucharest, Romania; camelia.diaconu@umfcd.ro; 5 Internal Medicine Department, Clinical Emergency Hospital of Bucharest, 105402 Bucharest, Romania; 6 Academy of Romanian Scientists, 050085 Bucharest, Romania; 7 Department of Pharmacy, Faculty of Medicine and Pharmacy, University of Oradea, 410028 Oradea, Romania

**Keywords:** acute coronary syndromes, systemic immune-inflammation index, mortality, major adverse cardiac and cerebrovascular events, biomarkers, STEMI, NSTEMI, inflammation

## Abstract

Background/Objectives: Inflammation plays a key role in acute coronary syndromes (ACS). The systemic immune-inflammation index (SII), which integrates immune and inflammatory markers, may serve as a valuable prognostic tool. This study aimed to evaluate the utility of SII as a short-term predictor of mortality and major adverse cardiovascular and cerebral events (MACCE) in ACS patients. Methods: A retrospective analysis was conducted on 964 ACS patients admitted in 2023. SII was calculated from admission hematological parameters. Primary and secondary outcomes were 30-day mortality and MACCE, respectively. Results: SII levels differed significantly across ACS subtypes (*p* < 0.001), highest in ST-segment elevation myocardial infarction (STEMI) and lowest in unstable angina. SII was markedly higher in deceased patients (2003.79 ± 1601.17) vs. survivors (722.04 ± 837.25; *p* < 0.001) and remained an independent predictor of mortality (OR = 1.038, *p* < 0.001). Similarly, SII was elevated in MACCE cases (1717 ± 1611.32) vs. non-MACCE (664.68 ± 713.11; *p* < 0.001) and remained predictive in multivariate analysis (OR = 1.080, *p* < 0.001). Predictive accuracy for MACCE was moderate (AUC = 0.762), improved when combined with GRACE 2, especially in specificity (*p* = 0.07). In STEMI, SII had excellent accuracy (AUC = 0.874), outperforming neutrophil–lymphocyte ratio and C-reactive protein. SII rose at 24 h and declined at 48 h in STEMI, with a slower decline in MACCE patients. Conclusions: SII proved to be a cost-effective biomarker reflecting inflammation, immunity, and thrombosis. Elevated SII predicted short-term MACCE and mortality in ACS, with improved prognostic power when combined with GRACE 2. Persistent elevation may signal ongoing inflammation and increased MACCE risk.

## 1. Introduction

Atherosclerosis is a significant cause of death worldwide [1]. Despite late advances in interventional revascularization and optimal drug therapy in acute coronary syndromes (ACS) [2], ACS continue to be associated with increased risk of mortality and morbidity [3]. Enhancing risk stratification in ACS is essential for prognostic improvement.

Atherosclerosis is characterized by low-grade chronic inflammation with episodes of acute exacerbations that can manifest clinically as ACS [4]. Immunity and inflammation play an essential role in every step of atherogenesis, from endothelial dysfunction to atherosclerotic plaque rupture [5,6]. The rupture of the unstable atherosclerotic plaque leads to platelet aggregation and thrombus formation with acute thrombotic obstruction of the coronary artery. This thrombogenic process is correlated with amplified inflammation, and, furthermore, the ischemic reperfusion injury of the cardiac muscle in patients with ACS is also influenced by inflammation [7].

White blood cells (WBCs) have been associated with increased risk of cardiovascular disease (CVD), myocardial infarction (MI), stroke [8,9,10], and all-cause mortality [11,12]. Neutrophils are studied as prognostic indicators in cardiovascular diseases and ACS [13,14]. Lymphocytes represent adaptive immunity that is implicated in the reduction of inflammation [15,16]. A low lymphocyte level is correlated with short-term mortality in MI [17].

Biomarkers combining two-line cells as the ratios of monocyte to lymphocyte (MLR), neutrophil to lymphocyte (NLR), and platelet to lymphocyte (PLR) have an even higher value as predictors of CVD and overall mortality [18,19,20,21]. In patients with ACS and chronic coronary syndromes (CCSs) undergoing percutaneous coronary intervention (PCI), elevated levels of NLR were correlated with worse prognosis. Furthermore, NLR was found to be correlated with the severity of coronary artery disease (CAD), as assessed by the Synergy Between PCI with TAXUS and Cardiac Surgery (SYNTAX) score [22]. Choi et al. have combined NLR with mean platelet volume, obtaining a better prediction of adverse outcome in patients with ACS [23]. A combination of NLR with PLR was shown to be a better prognostic indicator than each parameter alone in patients treated by primary PCI [24].

The systemic immune-inflammation index (SII) combines three blood cell subtypes (neutrophils, lymphocytes, and platelets) and captures the interaction between inflammation and the immune system response [25]. An increased level of SII was demonstrated to be correlated with a higher risk of CAD, with more severe CAD [26,27,28], poor coronary collateral circulation [29] and a higher risk of major cardiovascular events (MACEs) in heart failure patients [8], and following PCI [1,30,31] or cardiac surgery [32,33,34,35].

Various inflammatory biomarkers, such as interleukin-6, tumor necrosis factor-alpha, and C-reactive protein (CRP), were studied as prognostic indicators in ACS, although they are influenced not only by the ACS itself but also by comorbidities, as infection or malignancy [36,37]. The essential prognostic role of inflammation in atherosclerosis is further sustained by the improved outcome observed with anti-inflammatory therapy in trials like LoDoCo (Low-Dose Colchicine) and CANTOS (Canakinumab Anti-Inflammatory Thrombosis Outcome Study) [38,39].

Given the essential role of inflammation in the pathogenesis of ACS, the evaluation of the inflammatory process could predict the prognosis in ACS patients. Severe systemic inflammation is an indicator of poor prognosis and increased mortality in patients with ACS, but defining the best biomarkers that can reflect inflammation and guide cardiovascular treatment is still a challenge. The unbalanced activation of the innate and adaptive immunity results in platelet activation and thrombosis. The SII (calculated as neutrophil × platelet/lymphocyte) is an inflammatory index that contains elements from all the pathophysiological processes involved in ACS and can quantify the amount of inflammation. SII was studied in several diseases, such as cancer, chronic kidney disease (CKD), liver transplantation, and also in CVD [40,41] as an outcome predictor.

This research was conducted to assess the value of SII as a short-term predictor of mortality and major adverse cardiovascular and cerebral events (MACCE) in a large cohort of patients admitted for ACS. Patients with ST-elevation MI (STEMI) and patients treated with percutaneous interventional therapy were separately analyzed. The prognostic accuracy of SII was studied both independently and in combination with the validated Global Registry of Acute Coronary Events (GRACE) 2 risk score. Additionally, we compared the prognostic accuracy of several inflammatory biomarkers, including SII, NLR, and CRP, in predicting MACCE. Finally, we examined the evolution of SII at multiple time points in STEMI patients to evaluate trends in systemic inflammation and their association with MACCE.

## 2. Materials and Methods

### 2.1. Study Design

The present retrospective analysis included a cohort of 1109 patients consecutively admitted to the Cardiology Department of Bihor County Emergency Hospital, Oradea, Romania, over a period from January to December 2023. All individuals had confirmed diagnoses of ACS, which comprised cases of unstable angina (UA) as well as MI presentations, with and without ST-segment elevation.

Following the criteria established by the European Society of Cardiology (ESC), unstable angina (UA) was diagnosed in patients experiencing chest pain either at rest or during minimal physical exertion, in the absence of acute myocardial cell damage. Individuals were classified under UA if they exhibited rest angina lasting beyond 20 min, newly developed severe angina episodes, or a rise in angina frequency, duration, or occurrence during lower levels of exertion. Additionally, those with post-MI angina accompanied by ischemic changes on electrocardiogram (i.e., ST-segment depression or T-wave inversion/flattening), but without elevated high-sensitivity troponin I (hsTni), were included in this subgroup [42].

The diagnosis of MI was established according to the fourth universal definition, which mandates a dynamic change—either rise or fall—in hsTn concentrations, with at least one measurement exceeding the 99th percentile upper reference threshold. This biochemical criterion must be accompanied by at least one additional indicator, such as ischemic symptoms, novel ECG evidence of ischemia, pathological Q waves, or imaging findings revealing recent myocardial injury, including nonviable myocardium or regional wall motion defects identified via echocardiography, magnetic resonance imaging (MRI), or nuclear imaging compatible with ischemic etiology. Alternatively, coronary angiography revealing thrombus or autopsy findings can confirm the diagnosis [43].

MI cases were further categorized into STEMI or NSTEMI, depending on the presence of ST-segment elevation. The diagnostic cutoffs for ST elevation at the J point in two or more adjacent leads were stratified by age and sex: in leads V2–V3, elevations ≥ 2.5 mm for men under 40 years, ≥2 mm for men over 40, and ≥1.5 mm for women regardless of age; in all other leads, an elevation of ≥1 mm was considered significant. Furthermore, new-onset left bundle branch block fulfilling Sgarbossa’s criteria was also classified as STEMI [42,44].

**Figure 1:**
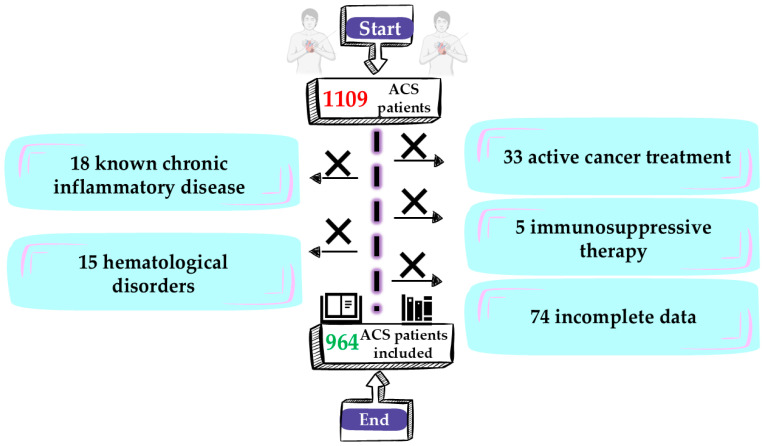
Flowchart depicting patient selection.

Subjects were excluded if they had pre-existing hematologic cancers (n = 15), were undergoing active oncologic therapies (n = 33), received immunosuppressive medications (n = 5), suffered from inflammatory or autoimmune disorders (n = 18), or presented incomplete clinical information (n = 74), as detailed in Figure 1.

After implementing the predefined inclusion and exclusion criteria, the final dataset comprised 964 patients. Because the study was retrospective, the cohort size was established by all qualifying admissions within the timeframe, thereby providing an extensive overview of clinical practice as it occurs in real-world settings.

Medical data was extracted from patients’ hospital records and the electronic database system. The variables collected included demographic characteristics such as age, sex, and smoking habits, alongside cardiovascular risk indicators like diabetes mellitus, hypertension, and dyslipidemia. Clinical measurements taken upon admission—heart rate, blood pressure, and body mass index (BMI)- were also documented. Risk assessment was performed using the GRACE 2.0 score, calculated through mdcalc.com, accessed on 5 June 2025 [45], incorporating eight parameters: clinical data (age, heart rate, systolic blood pressure, cardiac arrest on arrival, Killip classification) and laboratory findings (serum creatinine, elevated hsTni levels, and ST-segment deviations on ECG).

Laboratory results accessed from medical documentation reflected blood samples drawn at hospital entry, encompassing complete blood count (CBC), CRP, creatinine, hsTni, and cholesterol levels. In patients diagnosed with STEMI, serial CBC measurements recorded at admission, 24 h, and 48 h were analyzed to track changes in inflammatory biomarkers over time. The NLR was calculated as the quotient of the total neutrophil number over the lymphocyte count. Following this, the SII was computed by multiplying the NLR value by the platelet concentration [46]. CBC analyses were conducted utilizing the Alinity hq automated hematology analyzer (Abbott, Chicago, IL, USA), which uses Multi-Angle Polarized Scatter Separation technology to differentiate white blood cells and laser light scatter techniques for red blood cell and platelet assessment. The Alinity hq automated hematology analyzer undergoes routine calibration using manufacturer-provided calibration materials traceable to international reference standards. Possible variations are quickly detected and can be eliminated. Internal quality control is performed three times a day and whenever necessary with the help of control blood at three concentration levels: low, normal, and high. External quality assessment is conducted quarterly using a separate control product (control blood), which is processed in the same manner as a routine sample. The automated hematology analyzer requires calibration with calibration blood. Calibrations are performed annually, and when control values fall outside acceptable limits, with a calibrator (from Abbott, Chicago, IL, USA). The intra-assay coefficient of variation for most CBC parameters (including WBC, red blood cells, hemoglobin, hematocrit, platelets) typically ranges from 1% to 3% for normal samples, depending on the parameter and concentration. Inter-assay variability is controlled through regular maintenance, alignment checks, and participation in external quality assessment programs [47].

Hs-Tni levels were quantified by the Abbott Alinity hs-Tni assay. CRP concentrations were measured on the same Abbott Alinity platform via a turbidimetric assay.

In most cases (92%, or 667 out of 725), coronary angiography (CAG) was conducted using the radial artery as the access site. Prior to the procedure, every patient received a loading dose of 300 mg acetylsalicylic acid along with a P2Y12 receptor antagonist (either clopidogrel or ticagrelor), as well as unfractionated heparin 70–100 units per kilogram. Following the intervention, patients continued standard-of-care treatments for ACS, incorporating dual antiplatelet therapy combining aspirin with a P2Y12 inhibitor, alongside anticoagulant medications. Additional pharmacologic therapies such as beta-adrenergic blockers, angiotensin converting enzyme (ACE) inhibitors or angiotensin receptor blockers (ARBs), and potent statin regimens were initiated according to clinical judgment by the treating cardiologist.

The primary endpoint measure assessed was mortality within 30 days post-event. Secondary outcomes encompassed a composite endpoint of MACCE, including cardiac-related death, repeated episodes of non-fatal MI, strokes that did not result in death, newly diagnosed heart failure, and subsequent revascularization procedures. Clinical events and outpatient follow-up data were monitored and recorded for one month following the initial hospitalization.

A recurrent MI was identified when a new acute event followed the primary ACS episode, manifested clinically by recurring chest pain and evidenced on ECG by new ST-segment changes or novel pathological Q waves appearing in at least two adjacent leads. This was supported by rising hs-Tni levels confirmed through two sequential assays performed 3 to 6 h apart. In cases where the baseline hs-Tni had normalized, the standard criteria for diagnosing a new acute MI were applied. Conversely, if the initial hs-Tni remained elevated but was trending downward, a subsequent increase exceeding 20% was necessary to confirm the diagnosis of reinfarction [48].

Unplanned revascularization, following the initial PCI, was characterized as any repeated intervention imposed by the return of ischemic manifestations or a subsequent MI. This repeated intervention category comprised three distinct scenarios: (a) target lesion revascularization, indicating a repeated PCI addressing restenosis located at or within 5 mm of the originally treated lesion; (b) target vessel revascularization, referring to a new PCI performed unexpectedly on a different segment of the same coronary artery initially treated; and (c) unplanned revascularization involving a different coronary artery than that targeted during the initial procedure. Elective PCIs scheduled for finalizing the revascularization process were categorized as staged procedures and were excluded from the group classified as repeated revascularizations [49].

The diagnosis of heart failure was established by integrating clinical evaluation with findings obtained from transthoracic echocardiography, which was conducted by a cardiologist within the first 24 h after patient admission. Left ventricular systolic performance was assessed through the modified Simpson’s method, enabling precise calculation of the left ventricular ejection fraction (LVEF). Echocardiographic examinations were performed utilizing either the Siemens Acuson X300 system (Siemens Medical Solutions, Malvern, PA, USA) or the Philips CX50 point-of-care ultrasound device (Philips Healthcare, Taguig Philippines).

The diagnosis of stroke was based on neurological assessments corroborated by imaging studies, including cerebral computed tomography (CT) or MRI.

### 2.2. Statistical Analysis

Data management and statistical analyses were performed utilizing MedCalc version 19.4 (MedCalc Software Ltd., Ostend, Belgium) alongside SPSS version 25 (IBM Corp., Armonk, NY, USA). Quantitative data are expressed as mean values accompanied by their standard deviations (SDs), whereas qualitative data are summarized as frequencies and corresponding percentages. Table 1 summarizes the key statistical methods, software tools, significance criteria, and power analysis results employed in the study to ensure rigorous evaluation of clinical and laboratory data.

Given the limited event rates and suboptimal power in NSTEMI and UA groups, comprehensive multivariate analyses were confined to STEMI patients to ensure statistical reliability and valid conclusions.

### 2.3. Ethical Approval

This research adhered to the ethical principles outlined in the Declaration of Helsinki and received formal approval from the Ethics Committee of Bihor Clinical County Emergency Hospital, Romania (approval number 35967, dated 21 November 2024).

## 3. Results

A total of 964 patients admitted with ACS in the Bihor Clinical County Hospital from January 2023 to December 2023 were enrolled in the final analysis. In the entire cohort of patients, 380 (39.4%) patients were diagnosed with STEMI, 283 (29.4%) patients were diagnosed with NSTEMI, and 301 (31.2%) patients were diagnosed with UA. The mean age of the entire cohort population was 65.59 ± 11.76 years, and 621 (64.4%) patients were male. CAG was performed in 725 (75.2%) patients, and subsequently, 509 (52.8%) underwent interventional revascularization with stent implantation. Regarding the extent of CAD, CAG showed that 211 (30.6%) of them had one-vessel CAD, 153 (22.2%) had two-vessel CAD, 223 (32.3%) had three-vessel CAD, while 123 (12.8%) had no significant atherosclerotic stenosis (defined as >50–75% narrowing). SII values were not correlated with the extent of CAD, being 842.02 ± 995.48 in patients with one-vessel disease, 728.45 ± 66.89 in patients with two-vessel disease, 797.89 ± 839.68 in patients with three-vessel disease, and 586.34 ± 42.76 in patients without significant stenotic lesions (*p* = 0.164).

The mean SII levels increased significantly with the severity of ACS subtype (*p* < 0.001), with the highest values observed in patients with STEMI 1059.02 ± 1171.16, followed by NSTEMI 846.80 ± 1035.42, and the lowest in patients with UA 509.84 ± 444.00.

### 3.1. SII as a Predictor of Short-Term Mortality in ACS Patients

During hospitalization and within the first 30 days after ACS, a total of 76 patients (7.88%) died. Mortality was highest in the STEMI group, with 61 deaths (16.05%), compared to 11 deaths (3.88%) in the NSTEMI group and 4 deaths (1.32%) in the UA group. The baseline demographic, clinical, laboratory, and imaging parameters were compared between the groups of deceased patients and the patients who survived (Table 2). In the deceased group, the patients were older, and more commonly females. LVEF was significantly lower, and creatinine was significantly increased in the deceased group. Hematologic parameters (WBC, NLR, and SII) were significantly higher in patients who died, and hs-Tni and GRACE 2.0 risk score showed increased value in deceased patients. In the deceased group, patients had a more frequently diagnosis of STEMI.

PCI was performed less in the deceased group. Extensive CAD with three-vessel PCI was also more frequent in patients who died. Among ACS patients who did not undergo PCI, there were three main groups:

- Patients who did not undergo CAG; this included older, frail individuals with severe comorbidities, those who refused the procedure, and those who died before it could be performed.
- Patients with extensive CAD who could not be treated with PCI and were instead referred for CABG.
- Patients without significant coronary artery stenosis (defined as less than 50–75% narrowing).

The first two categories (no CAG and referred for CABG) were more commonly found among those who died (the deceased group), whereas the third category (no significant stenosis) was more frequently observed in those who survived.

**Figure 2:**
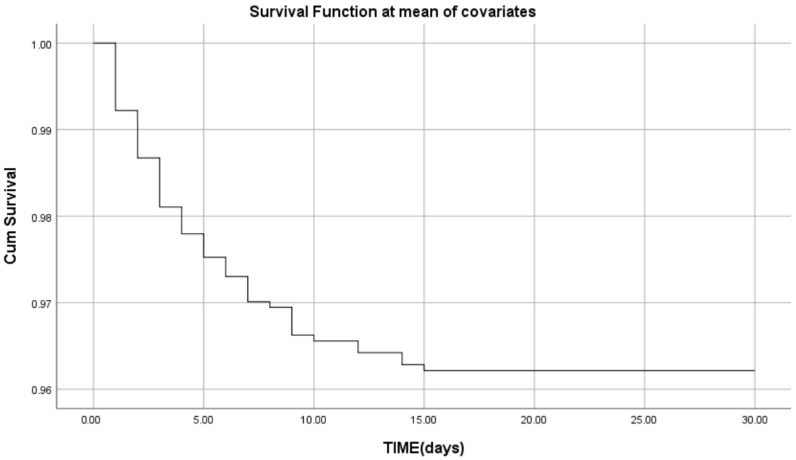
Survival curve. Cox regression multivariate analysis.

In Cox regression analysis, after rescaling SII (expressed as per 100-unit increase) to improve interpretability, SII persisted as an independent predictor of mortality (OR = 1.39; 95% CI: 1.232–1.562; *p* < 0.001). Other parameters that proved independent predictors were age OR 1.043 (95% CI 1.019–1.067), *p* < 0.001, LVEF OR = 0.939 (95% CI 0.917–0.962), *p* < 0.001, creatinine OR = 1.33 (95% CI 1.11–1.59), and troponin OR = 1.002 (1.001–1.007), *p* = 0.001. The survival curve revealed that the survival probability was good, decreasing only slightly below 95% by the end of the observation period, and most of the events occurred in the early period (Figure 2).

**Figure 3:**
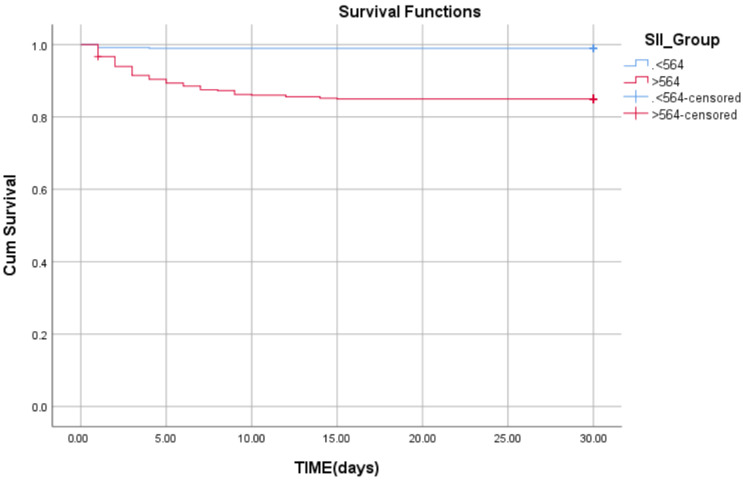
Kaplan–Meier survival curve in patients with low SII (<564) versus patients with high SII (>564).

A Kaplan–Meier survival curve was plotted after dividing patients into two groups based on the median SII value of 564. Patients with an SII ≤ 564 were classified as the low SII group, while those with an SII > 564 were classified as the high SII group. There was a significant difference in survival distribution between patients with high SII, who were associated with increased mortality risk, and patients with low SII in whom the risk of mortality is significantly decreased (log-rank Mantel–Cox test χ^2^ = 63.751, *p* < 0.001) (Figure 3).

### 3.2. SII as a Predictor of MACCE in ACS Patients

MACCE, including cardiac death, recurrent non-fatal MI, non-fatal stroke, new-onset heart failure, and repeated revascularization, were recorded in 147 patients (15.25%). Heart failure was present in 116 patients (12%), recurrent MI and repeated revascularization in 17 (1.8%) patients, repeated revascularization for angina in 4 patients (0.41%), and stroke in 7 patients (0.7%). Multiple events in the same patient were registered just once. MACCE were registered predominantly in the STEMI subgroup, 98/380 (25.79%). In the NSTEMI subgroup, there were 36/283 (12.72%) and in the UA subgroup, there were 13/301 (4.31%) MACCE. Demographic, clinical, and paraclinical parameters in patients with and without MACCE are depicted in Table 3.

### 3.3. The Discriminative Value of SII as a Predictor of MACCE in ACS Patients. Correlation with GRACE 2 Risk Score

**Figure 4:**
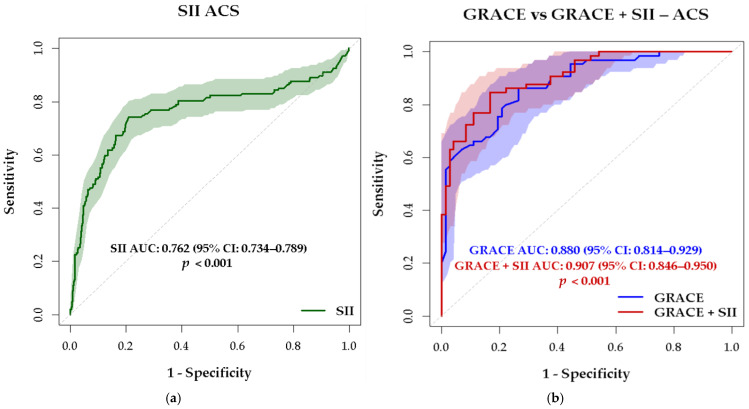
ROC curves for (**a**) SII as a predictor of MACCE and (**b**) a combined parameter SII and GRACE 2 risk score versus only GRACE 2 risk score as a predictor of MACCE in ACS patients.

SII had a significant bivariate direct correlation with GRACE 2 risk score (r = 0.482, *p* < 0.001), with MACCE occurrence (r = 0.308, *p* < 0.001), the diagnosis of STEMI (r = 0.206, *p* < 0.001), and heart failure (r = 0.377, *p* < 0.001) and an inverse correlation with LVEF (r = −0.229, *p* < 0.001). ROC curve analysis demonstrated that SII is a significant predictor of MACCE, yielding an area under the curve (AUC) of 0.762 (95% CI: 0.734–0.789; *p* < 0.001) (Figure 4a). At a cut-off value greater than 867, the sensitivity was 74.15% and the specificity was 78.95%. GRACE score was confirmed as an important predictor of MACCE with an AUC of 0.880 (95% CI 0.814–0.929, *p* < 0.001). An optimal cut-off value >133 demonstrated a sensitivity of 85.71% and a specificity of 76.12%.

For a combined parameter GRACE 2 score and SII, ROC curve analysis revealed an AUC of 0.907 (95% CI 0.846–0.950), with an increased specificity 91.04%, sensitivity 77.14%, but the improvement of AUC for the combined parameter compared to AUC for GRACE 2 score did not reach statistical significance (*p* = 0.07) (Figure 4b). The combined score (SII and GRACE 2) significantly increases specificity, respectively, the ability to rule out patients unlikely to experience MACCE.

Of 509 ACS patients (52.8%) who underwent PCI with stent implantation, 60 (11.79%) developed MACCE. The highest event rate was observed in the STEMI subgroup (16.23%, 44/271), followed by NSTEMI (8.94%, 11/123) and UA (4.34%, 5/115).

**Figure 5:**
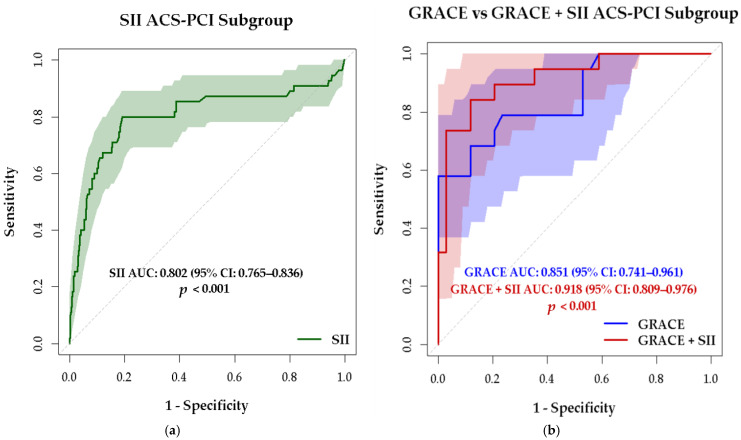
ROC curves for (**a**) SII as a predictor of MACCE and (**b**) a combined parameter SII and GRACE 2 risk score versus GRACE 2 risk score as a predictor of MACCE in ACS patients treated with PCI.

ROC curve analysis demonstrated that in patients who underwent interventional revascularization, the SII predicted MACCE with an AUC of 0.802 (95% CI: 0.765–0.836), *p* < 0.001, showing 80% sensitivity and 80.84% specificity at a cut-off value of 865 (Figure 5a). The GRACE 2 score showed predictive value for MACCE with an AUC of 0.851 (95% CI: 0.741–0.961), *p* < 0.001, achieving 57.89% sensitivity and 100% specificity at a cut-off value greater than 154.

For a combined parameter SII and GRACE 2, the AUC is increasing to 0.918 (95% CI 0.809–0.976), *p* < 0.001, sensitivity increased to 89.47% and specificity was 88.24%; compared with the AUC for GRACE only, the improvement does not reach statistical significance (*p* = 0.13) (Figure 5b).

### 3.4. SII as a Predictor of MACCE in STEMI Patients

Among 380 patients diagnosed with STEMI, 98 developed MACCE, including 43 in the group that underwent primary PCI (PPCI) and 55 in the group without interventional revascularization. Table 4 presents clinical, paraclinical, and demographic data in STEMI patients with and without MACCE.

Older age, diabetes, female sex, and higher GRACE scores were significantly more common in patients with MACCE. SII was more than 2.4 times higher in MACCE patients. Other inflammatory markers (NLR, CRP, and WBC) were also significantly elevated, sustaining the role of systemic inflammation as a predictor of adverse events in STEMI. Lower LVEF and higher creatinine were common in patients with MACCE. Patients not treated with PCI developed MACCE more frequently. MACCE was also more frequent in patients not amenable to PCI, due to more severe or multivessel disease.

**Figure 6:**
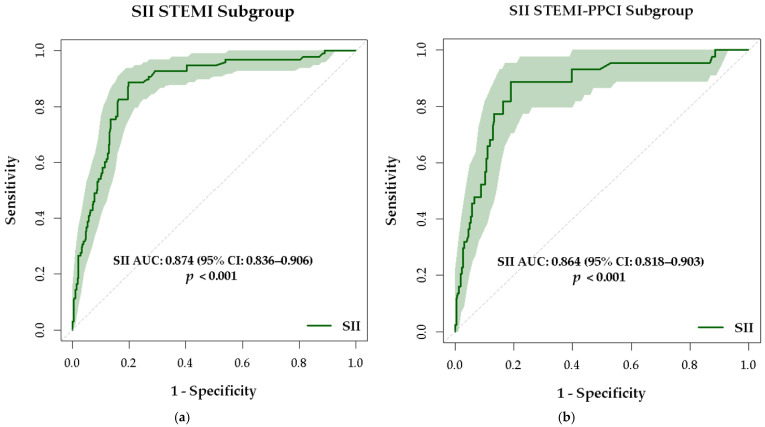
ROC curves for (**a**) SII as a predictor of MACCE in STEMI patients and (**b**) SII as a predictor of MACCE in STEMI patients with PPCI.

In multiple regression analysis, an increased SII was an independent predictor of MACCE in patients with STEMI. When expressed per 100-unit increase, SII was associated with a significantly increased risk of MACCE, OR 2.69 (95% CI: 1.92–3.76, *p* < 0.001), highlighting the clinical relevance of elevated SII values in this population. Other independent predictors for MACCE remained: creatinine OR 3.4 (95% CI 1.063–11.11), *p* = 0.03, a low LVEF (OR 0.91 (95% CI 0.86–0.96), *p* = 0.001 and CRP 1.081 (85% CI 1.02–1.13), *p* = 0.003. SII showed excellent discriminatory power in STEMI patients. The accuracy of SII in predicting MACCE was good in patients with STEMI, with an AUC of 0.874 (95% CI 0.836–0.906), *p* < 0.001, for a cut-off value >866, sensitivity 88.78%, and specificity 80.14% (Figure 6a). The ability of SII in detecting the patients at risk for MACCE is increased, while specificity is also good, making SII a reliable standalone predictor in STEMI patients. SII is maintaining its performance in the subgroup of STEMI patients who underwent interventional revascularization. In the STEMI subgroup who underwent PPCI, the SII showed strong predictive value for MACCE, with an AUC of 0.864 (95% CI: 0.818–0.903), *p* < 0.001. Using a cut-off value greater than 865, SII achieved a sensitivity of 88.64% and a specificity of 81.06% (Figure 6b). While the AUC is slightly lower compared to the general STEMI group, it still indicates a good accuracy, with almost identical sensitivity and slightly increased specificity.

### 3.5. Comparison of Inflammatory Markers in Predicting MACCE in ACS Patients

All analyzed inflammatory markers (SII, NLR, and CRP) were significantly higher in patients with adverse events. In patients with MACCE versus patients without MACCE, SII was 1717 ± 1611.32 vs. 664.68 ± 713.11 (*p* < 0.001), NLR was 8.76 ± 5.79 vs. 3.81 ± 3.79 (*p* < 0.001), and CRP was 8.84 ± 11.59 vs. 3.23 ± 5.1 (*p* < 0.001).

To assess the discriminatory ability for each inflammatory marker as a MACCE predictor in ACS, ROC curve analysis was performed. The AUC for SII as a predictor of MACCE revealed a moderate discriminatory power—0.762 (95% CI 0.734–0.789), *p* < 0.001, while NLR and CRP had a fair discriminative power, with an AUC of 0.691 (95% CI: 0.611–0.722, *p* < 0.001) for NLR and an AUC of 0.670 (95% CI: 0.590–0.749, *p* < 0.001) for CRP.

Comparison of inflammatory markers in ACS patients treated with PCI revealed significantly increased values in patients with MACCE versus patients without MACCE. SII was 1851.06 ± 1667.74 vs. 645.60 ± 593.72 (*p* < 0.001); NLR was 7.60 ± 5.64 vs. 3.79 ± 3.41 (*p* < 0.001), and CRP was 9.06 ± 13.6 vs. 3.29 ± 5.05 (*p* = 0.02). ROC curve analysis for ACS patients treated with interventional revascularization revealed that SII had the best predictive power for MACCE with an AUC of 0.802 (95% 0.765–0.836), *p* < 0.001. NLR and CRP had a fair discriminative ability with an AUC of 0.669 (95% CI 0.545–0.793), *p* = 0.002, and, respectively, 0.645 (95% CI 0.528–0.763), *p* = 0.009.

In STEMI patients with MACCE, inflammatory markers were significantly increased. SII was 2042.16 ± 1630.77 vs. 717.37 ± 691.17 (*p* < 0.001), NLR was 9.31 ± 5.87 vs. 5.12 ± 4.56 (*p* < 0.001), and CRP was 12.6 ± 13.2 vs. 4.7 ± 6.1 (*p* = 0.001). ROC curve analysis revealed an excellent predictive power of SII for MACCE with an AUC 0.874 (95% CI 0.836–0.906), *p* < 0.001, while NLR and CRP had a fair predictive value with AUC 0.695 (95% CI 0.589–0.801), *p* = 0.001, respectively, AUC 0.691 (95% CI 0.585–0.796), *p* = 0.004.

STEMI patients treated with PPCI, who developed MACCE, had increased values of inflammatory markers. SII was 2143.59 ± 1721.03 vs. 723.94 ± 727.21 (*p* < 0.001), NLR was 7.81 ± 5.18 vs. 4.90 ± 4.25 (*p* = 0.001), and CRP was 11.46 ± 14.81 vs. 4.22 ± 5.36; *p* = 0.02. ROC curve analysis revealed excellent discriminative power for SII with an AUC of 0.864, (95% CI 0.818–0.903), *p* < 0.001 versus reduced discriminative power for NLR AUC 0.665 (95% CI 0.528–0.802), *p* = 0.011 and not significant for CRP AUC 0.618 (95% 0.474–0.762), *p* = 0.06.

### 3.6. Serial SII Assessment in Patients with STEMI

SII levels changed significantly over time during hospitalization. SII mean values are presented in the general group of STEMI patients and in the subgroup treated with PPCI (Table 5).

Pairwise comparisons of SII measured at three different time points: baseline, 24 h, and 48 h in STEMI patients and in the PPCI subgroup, using repeated measures ANOVA (General Linear Model) are revealed in Table 6. SII levels changed significantly for 48 h, and this was revealed by the significant main effect of time on SII values (Wilks’ Lambda = 0.631, *p* < 0.001).

In both subgroups, SII registered a peak at 24 h, followed by a decrease at 48 h. These findings highlight that SII is a dynamic inflammation marker that peaks with acute MI and declines during recovery. The SII levels at baseline and 24 h were comparable between groups, but in the STEMI-PPCI subgroup, a slightly increased residual SII was detected at 48 h, probably connected to the postprocedural inflammation related to stent implantation. These results underscore the utility of serial SII measurement with the aim of monitoring the inflammatory response, while the SII temporal trend may differ slightly in correlation with the interventional treatment.

For assessment of temporal SII trends and their association with outcomes in STEMI patients, SII levels at baseline, 24 h, and 48 h were compared between those who developed MACCE versus those who did not (Table 7). A significant time × MACCE interaction was observed (Wilks’ Lambda = 0.915, *p* < 0.001), suggesting that the evolution of SII over time differed significantly between patients who developed MACCE and those who did not.

Patients who developed MACCE had nearly 3-fold higher SII at baseline than those who did not, which indicates an important predictive value of baseline SII for adverse events.

In STEMI patients without MACCE, SII peaks at 24 h, then declines sharply by 48 h, suggesting a resolving inflammatory response. In patients with MACCE, SII remains persistently elevated, with only a moderate decline by 48 h.

In the STEMI-PPCI subgroup, a very similar trend was observed; the MACCE group had much higher SII at all time points and SII declined more slowly, indicating persistent inflammation, which may be more intense following interventional treatment. Multivariate tests confirm significance with Wilks’ Lambda = 0.574, *p* < 0.001, partial eta squared = 0.426, indicating a large effect size. The MACCE–Time interaction in STEMI PPCI patients is depicted in Table 7.

Discriminative power was compared between SII measured at baseline, 24 and 48 h as predictors of MACCE. Baseline SII and SII at 24 h both showed excellent diagnostic performance. SII at 48 h remains significant, but less performant, indicating that early values of SII might be more reliable predictors (Table 8).

## 4. Discussion

Across this sizable cohort of ACS patients, the biomarker SII significantly increased in parallel with disease severity: the highest values were observed in STEMI, followed by NSTEMI, and the lowest in UA. This gradient in SII values reflects the degree of systemic inflammation and myocardial injury, which is higher in STEMI, moderate in NSTEMI, and lowest in UA. Furthermore, SII predicts short-term mortality. Patients who died during hospitalization or within 30 days had significantly higher SII levels than survivors. The role of SII as an independent predictor of short-term mortality was confirmed in Cox regression analysis, reinforcing its value as a prognostic biomarker.

SII was also significantly increased in the group of ACS patients who experienced MACCE (a combined end point of death, non-fatal MI or stroke, repeat revascularization, heart failure). In multivariate logistic regression, SII was independently associated with MACCE, enhancing its clinical utility in risk stratification. Significant correlations were observed between the SII and several clinical parameters. SII showed a moderate positive correlation with the GRACE 2 severity score. Additionally, positive correlations were found between SII and MACCE, STEMI diagnosis, and heart failure. Conversely, SII was inversely correlated with LVEF.

In patients with ACS, the levels of inflammatory biomarkers (SII, NLR, and CRP) were significantly elevated in those who developed complications. ROC curve analysis showed that SII is the strongest predictor, outperforming both NLR and CRP, especially in the subgroup of ACS patients treated with interventional revascularization. These findings reveal the role of systemic inflammatory markers, particularly SII, in short-term risk stratification of ACS patients.

In the subgroup of STEMI patients, including patients treated with primary PCI, increased levels of SII, NLR, and CRP were associated with the occurrence of MACCE. ROC curve analysis demonstrated that SII had the highest predictive accuracy, compared to NLR and CRP. These findings sustain SII as a strong, independent inflammatory biomarker for early risk stratification in STEMI patients.

In both STEMI and STEMI-PCI patients, SII values followed a similar dynamic trend, rising significantly at 24 h post-admission before declining at 48 h. A significant MACCE–Time interaction was observed. In both the overall STEMI cohort and the PCI-treated subgroup, SII levels were significantly higher at all time points in patients who developed MACCE compared to those who did not. Patients who developed MACCE had nearly 3-fold higher SII at baseline than those who did not, which indicates a strong predictive value of baseline SII for adverse events. SII levels decreased over time in both groups, but the decline was substantially less pronounced in the MACCE group, particularly in the STEMI-PCI subgroup, suggesting that persistent systemic inflammation may be more prominent following interventional treatment. A persistent increase in SII may reflect ongoing systemic inflammation and worse short-term outcomes. Repeated measurements of inflammatory markers may better reflect the persisting inflammation and can provide superior prognostic data.

SII alone had moderate accuracy in predicting MACCE in a large cohort of ACS patients. Combining SII with GRACE 2 may help improve specificity, but lacks definitive statistical significance. SII has a good discriminative power in the subgroup of ACS patients treated with PCI, slightly improved compared to the general cohort, with both high sensitivity and specificity. The combined parameter (SII and GRACE 2) had very good overall accuracy as a MACCE detector in ACS patients treated with interventional revascularization, with a significantly increased sensitivity, but the increase in AUC versus GRACE 2 alone was not statistically significant. There is a potential additive value in combining inflammatory markers with established clinical scores for risk stratification. In STEMI patients, SII proved to be a strong independent marker for MACCE risk. Its predictive power was maintained in STEMI subgroups undergoing primary PCI, confirming its potential usefulness in the setting of interventional treatment.

SII has emerged as an independent and robust predictor of cardiac mortality and poor outcome in patients with ACS in several other studies [46,51,52,53,54,55]. A previously published study assessed the prognostic value of SII in ACS patients, regardless of age or treatment modality, and compared it with established severity scores. Their findings were consistent with ours, demonstrating significantly higher SII levels in patients with STEMI compared to those with NSTEMI or UA. Moreover, SII showed significant associations with Killip class, TIMI score, and GRACE 2.0 score, and outperformed both NLR and WBC in predicting MACE and mortality [46].

In another research performed by Su et al., a retrospective real-world study in which the authors used the Multiparameter Intelligent Monitoring in Intensive Care (MIMIC) III database, it was demonstrated that a high SII was an independent predictor of short- and long-term all-cause mortality in ACS [51].

The inflammatory marker SII was identified as an independent prognosticator of MACE in patients with newly diagnosed ACS in the research of Gao Yi et al. [52]. Elevated SII levels remained significantly associated with adverse outcomes, including non-fatal MI, heart failure, and the need for repeated revascularization, despite adjusting for additional cardiovascular risk variables. Adding SII to existing conventional risk factor models significantly improved the predictive value for MACEs in patients with initially diagnosed ACS [52].

The study of Shi et al. investigated whether the inflammatory biomarker SII can independently predict prognosis in patients with ACS and CKD [53]. Elevated SII levels remained independently associated with MACEs, all-cause mortality, cardiovascular death, non-fatal MI, and congestive heart failure in patients with ACS and CKD, even after adjusting for age and conventional cardiovascular risk factors [53].

Data from the NOAFCAMI-SH registry, which investigates new-onset atrial fibrillation following acute MI in Shanghai, demonstrated a clear association between elevated SII and both myocardial damage and impaired cardiac function post-MI. Moreover, increased SII levels were linked to a greater risk of all-cause and cardiovascular mortality, serving as an independent prognostic factor for long-term death, specifically in diabetic patients [56]. In our cohort of patients, we also observed a significant association between an increased SII and hs-Tni, and between SII and new onset heart failure, and an inverse correlation with LVEF, SII being an indicator of a more extensive myocardial necrosis and in consequence, of a more depressed systolic function and increased frequency of heart failure.

Different results were observed by Dziedzic et al. [6] in a study where the association between the severity of inflammation determined by SII and CAG-determined extent of CAD was analyzed in patients with ACS or stable CAD. Although patients with ACS exhibited significantly increased SII values overall, in the subgroups of STEMI, NSTEMI, and UA, the values were similar. Additionally, no significant correlation was identified between SII and the extent of CAD.

Different results were observed in other studies, with a positive correlation described between SII and the severity of CAD [26,28,52], while no correlation with the extent of CAD was observed in the present study. The absence of a significant association between the SII and the extent of CAD may be attributed to the distinct pathophysiological processes these parameters reflect. SII reflects systemic inflammation and is associated with plaque vulnerability, endothelial dysfunction, and the acute thrombotic environment that contributes to clinical events such as MACCE. In contrast, the angiographic extent of CAD (defined by the number of affected vessels) primarily reflects the chronic atherosclerotic burden rather than the active inflammatory state or plaque instability. Therefore, although SII may be a strong predictor of acute events, its correlation with the anatomical extent of CAD may be limited. The inconsistency with earlier studies that reported a positive correlation between SII and CAD burden may also be explained by differences in study populations and baseline cardiovascular risk profiles. While the current study included patients with ACS, studies by Erdogan et al. [26] and Candemir et al. [28] focused on patients with CCS. Furthermore, the definition used to define CAD burden varied: while the current study used the number of diseased vessels, previous studies employed severity scoring systems such as the SYNTAX score [26] or assessed functionally significant coronary lesions using fractional flow reserve measurements [28].

Although inflammation has an important role in diabetes mellitus and atherosclerosis [57], a study performed by Tuzimek A et al., which evaluated the utility of inflammatory markers including the SII, systemic inflammation response index (SIRI), and aggregate index of systemic inflammation (AISI) in patients with ACS and CCS with either diabetes or prediabetes, found no significant correlations between ACS and the levels of SII, SIRI, or AISI [58]. A recent large real-world cohort study found similar 1-year outcomes for STEMI and NSTEMI patients. However, diabetes and incomplete revascularization significantly impacted results. In diabetics, diffuse atherosclerosis, microvascular dysfunction, and inflammation increase the risk of recurrent ischemic events, particularly when revascularization is incomplete or staged [59].

The most effective reperfusion strategy for managing acute STEMI is primary PCI. Despite substantial advancements in revascularization techniques and improved patient prognosis, adverse cardiovascular outcomes still occur, with mortality rates ranging from 4% to 12% in STEMI patients [60]. Several clinical factors can influence the outcome following primary PCI, including patient age, a prior MI, Killip classification, and the extent of CAD [61]. Inflammation also plays a critical role in the initiation, progression, and complications development in acute MI. Atherosclerotic plaque rupture and consecutive thrombosis, the main pathophysiological events involved in acute MI, are a consequence of inflammation and oxidative stress [62]. Inflammation and thrombosis have important roles in the initiation and progression of STEMI [63]. Furthermore, during and after PCI, the implantation of a stent stimulates endothelial activation and inflammatory response. Platelets and leukocytes are key elements in atherosclerosis and ACS. After activation, platelets release inflammatory cytokines that create a prothrombotic status [64]. An increased number of neutrophils is associated with larger infarcts, complications, MACE, and mortality [65]. Neutrophils release neutrophil extracellular traps that can be detected in atherosclerotic plaque. Lymphocytes control the immune response, alleviate inflammation, and diminish myocardial damage. A low level of lymphocytes is correlated with an elevated risk of mortality and MACE [66].

In our study population with ACS, the subgroup of patients that was treated with PCI and developed MACCE had significantly higher SII values, SII being an independent predictor of MACCE. In the subgroup of patients with STEMI, SII had a good accuracy in predicting MACCE, including the subgroup of patients treated by primary PCI. ROC curve analysis demonstrated that SII had the highest predictive accuracy, outperforming NLR and CRP. These findings underscore SII as a strong, independent inflammatory biomarker for early complication risk stratification in STEMI patients.

It has been shown in several studies that SII is a predictor of mortality and MACE in ACS patients undergoing PCI [25,51,55]. Huang et al. [25] showed that patients older than 65 years who underwent PCI with higher SII had a higher mortality. The retrospective study of Saylik et al. on more than 800 patients with STEMI revealed that SII can independently predict cardiovascular death and MACE, including nonfatal MI and stroke, congestive heart failure, and revascularization in patients with STEMI after primary PCI [67]. Incorporating the SII into a model with other cardiovascular risk factors, such as hypertension, diabetes, age, and male gender, enhanced the predictive accuracy for cardiovascular events. SII was a better predictor of MACE compared to NLR and PLR [67].

Other studies have observed that SII can predict the no-reflow phenomenon following PCI in STEMI patients [68,69]. In a study by Omar et al. [70], SII, along with the SYNTAX score and TIMI risk score, was recognized as a standalone prognostic factor for mortality within one year among STEMI patients treated with PPCI. These results suggest that SII may function as an important indicator in risk stratification, complementing established risk scores such as SYNTAX II and TIMI.

A retrospective study conducted by Liu et al. investigated whether dynamic changes in the SII following PPCI in patients with STEMI are associated with cardiovascular outcomes [71]. The inflammatory response is amplified during PCI, and concomitantly used drug therapy with antiplatelets and statins can decrease inflammation. In consequence, monitoring temporal changes in SII may more accurately reflect the dynamic inflammatory process in these patients. The study revealed that the SII measured 12 h after-PPCI and at 30 days after hospital discharge was a predictor of short- and long-term outcomes. Tracking serial variations in SII enables identification of patients exhibiting amplified inflammatory activity who may benefit from specialized therapeutic strategies [71].

In our cohort for both STEMI and STEMI-PPCI patients, SII values followed a similar dynamic trend, rising significantly at 24 h post-admission before declining at 48 h. A significant MACCE time interaction was noted. In both the overall STEMI cohort and the PCI-treated subgroup, SII levels were significantly higher at all time points in patients who developed MACCE. In the MACCE group, baseline SII was 3-fold higher and SII remained persistently elevated, with only a moderate drop by 48 h. Furthermore, SII at baseline and 24 h had excellent discriminative power for the occurrence of MACCE.

Despite all the progress in the management of ACS, the morbidity and mortality remain increased in ACS, the risk of mortality being five to six times higher in people who experience recurrent events [72]. Considering the increased risk of ACS patients, accurate risk stratification is essential for better management and improved outcomes. Various biomarkers and risk scores have been studied as prognostic indicators [42].

Due to the important role of inflammation in the development, progression, and destabilization of atherosclerotic plaque, biomarkers that reflect inflammation may aid in the improvement of risk evaluation [73]. In the destabilization of atherosclerotic plaque, the innate and adaptative immunity have a complex role. Composite inflammatory markers more comprehensively reflect inflammation.

The SII is a simple marker of the inflammatory and immune status, which is derived from the three cell types (neutrophils, platelets, and lymphocytes). In our study, SII was found to be an independent predictor of MACCE and death in patients with ACS. The predictive accuracy was good, especially in patients with STEMI, including the subgroup with PPCI. The value of SII gradually increased from UA to STEMI patients, reflecting the intensity of the inflammatory process. Although SII had an important correlation with GRACE 2 risk score, combining both parameters slightly improved predictive accuracy without reaching statistical significance. The accuracy of SII proved to be superior compared to other biomarkers such as NLR and CRP. In clinical practice, determining SII could help in the risk assessment of ACS patients, especially in the setting of STEMI. A slowly declining SII may be an indicator of persistent inflammation and a high risk of MACCE. These results suggest that both baseline and dynamic inflammatory biomarkers may help in short-term risk stratification in STEMI patients.

The limitations of the study derive from its retrospective design; selection bias cannot be excluded, and secondly, patients were followed up for one month, long-term prognostic significance of inflammatory markers not being evaluated.

## 5. Conclusions

SII is a widely accessible biomarker that captures the complex interplay of inflammation, immune response, and thrombosis characteristic of ACS. Elevated levels of SII are good predictors of short-term MACCE and mortality in ACS patients. SII has the potential to identify high-risk individuals with ACS and can aid in risk stratification. Our research proved that the accuracy of SII in the prediction of MACCE was good and was improved when the biomarker was combined with the GRACE 2 risk score, but the improvement did not reach statistical significance. Persistently high or slowly declining SII may indicate sustained inflammation and a higher risk for MACCE.

## Figures and Tables

**Table 1:** Overview of the statistical approaches used.

Aspect	Details
Software used	SPSS v. 25 (IBM Corp.), MedCalc v. 19.4 (MedCalc Software), R Core Team, (Vienna, Austria) (2025)
Data presentation	Continuous variables: mean ± SD; categorical variables: frequencies and percentages
Test for continuous variables	Independent samples *t*-test
Test for categorical variables	Chi-square test
Statistical significance	*p* value < 0.05
Predictive accuracy assessment	ROC curve analysis with AUC calculation
Cutoff determination	Cutoffs based on Youden index maximizing sensitivity and specificity
Multivariate modeling	Binary logistic regression for independent predictors
Mortality analysis	Multivariable Cox proportional hazards regression
Temporal SII variation	Repeated measures ANOVA (General Linear Model) with Bonferroni correction
Power analysis tool	Post hoc using G*Power version 3.1 with α = 0.05, OR ≥ 1.5 [50]
Overall cohort power (n = 946)	99%, event rate: 15.25%
STEMI subgroup power (n = 380)	87%, event rate: 25.79%
NSTEMI subgroup power (n = 283)	77%, event rate: 12.23%
UA subgroup power (n = 301)	77%, event rate: 4.31%
Limitations in subgroups	Power below 80% limits multivariate modeling reliability
Subgroup analysis decision	Detailed analysis restricted to STEMI subgroup for robustness and power

SPSS, statistical package for the social sciences; SD, standard deviation; ROC, receiver operating characteristic; AUC, area under the curve; SII, systemic immune-inflammation index; ANOVA, analysis of variance; General Linear Model; major adverse cardiac and cerebrovascular events; OR, odds ratio; acute coronary syndrome; STEMI, ST-elevation myocardial infarction; NSTEMI, non-ST-elevation myocardial infarction; UA, unstable angina.

**Table 2:** Demographic, clinical, and paraclinical parameters in survivors and deceased ACS patients.

Parameters	Total (n = 964)	Deceased (n = 76)	Survivors (n = 888)	*p*
Age (years)	65.59 ± 11.758	73.39 ± 11.775	64.91 ± 11.51	<0.001 *
Sex (M)	621/964 (64.42%)	36/76 (47.37%)	585/888 (65.88%)	0.002 *
Smoking	232/964 (24.06%)	8/76 (10.52%)	224/888 (25.22%)	0.004 *
Hypertension	650/964 (67.43%)	23/76 (30.26%)	627/888 (70.60%)	<0.001 *
DM	308/964 (31.95%)	29/76 (38.15%)	279/888 (31.41%)	0.22
BMI (kg/m^2^)	30.95 ± 5.31	29.053 ± 4.65	31.116 ± 5.33	0.09
LVEF (%)	45.96 ± 9.45	34.25 ± 9.47	46.97 ± 8.75	0.001 *
Creatinine (mg/dL)	1.09 ± 0.64	1.79 ± 1.11	1.03 ± 0.55	
CRP (mg/L)	3.99 ± 6.70	12.35 ± 9.4	3.57 ± 6.26	
WBC (×10^3^/μL)	10.83 ± 4.59	14.75 ± 7.06	10.46 ± 4.10	
NLR	4.57 ± 4.52	10.63 ± 6.01	4.05 ± 3.97	
SII	825.24 ± 983.85	2003.79 ± 1601.17	722.04 ± 837.25	
hs-Tni (pg/mL)	1627.92 ± 2849.621	4623.42 ± 5884.92	1367.89 ± 2237.818	<0.001 *
GRACE 2	139.12 ± 41.89	175.28 ± 32.43	118.34 ± 31.29	
STEMI	380/964 (39.41%)	61/76 (80.26%)	319/888 (35.92%)	
NSTEMI	283/964 (29.36%)	12/76 (15.79%)	271/888 (30.52%)	0.006 *
UA	301/964 (31.22%)	4/76 (5.26%)	297/888 (33.44%)	<0.001 *
Cholesterol (mg/dL)	176.98 ± 47.56	164.83 ± 44.64	178.04 ± 47.69	0.88
PCI	509/964 (52.80%)	32/76 (42.11%)	477/888 (53.72%)	0.05 *
One vessel	211/509 (41.45%)	8/32 (25%)	203/477 (42.56%)	0.02 *
Two vessels	153/509 (30.5%)	4/32 (12.5%)	149/477 (31.24%)	0.012 *
Three vessels	145/509 (28.48%)	20/32 (62.5%)	125/477 (26.21%)	0.005 *
No PCI	455/964 (47.19%)	44/76 (57.90%)	411/888 (46.28%)	0.02 *
No CAG	239/455 (52.52%)	29/44 (65.91%)	210/411 (51.09%)	0.03 *
Not amenable to PCI	93/455 (20.44%)	15/44 (34.09%)	78/411 (18.98%)	0.018 *
No significant lesions	123/455 (27.03%)	0	123/411 (29.93%)	<0.001 *

M, male; DM, diabetes mellitus; BMI, body mass index; LVEF, left ventricular ejection fraction; CRP, C reactive protein; WBC, white blood cells; NLR, neutrophil to lymphocyte ratio; SII, systemic immune-inflammation index; hs-Tni, high-sensitivity troponin i; GRACE 2, Global Registry of Acute Coronary Events 2 Score; STEMI, ST elevation acute myocardial infarction; NSTEMI, non-ST elevation myocardial infarction; UA, unstable angina; PCI, percutaneous coronary intervention; CAG, coronary angiography; * *p* < 0.05, statistically significant.

**Table 3:** Demographic, clinical, and paraclinical parameters in patients with and without MACCE.

Parameters	Total (n = 964)	MACCE (n = 147)	Non-MACCE (n = 817)	*p*
Age (years)	65.59 ± 11.758	70.81 ± 11.572	64.65 ± 11.550	<0.001 *
Sex (M)	621/964 (64.4%)	82/147 (55.78%)	539/817 (65.97%)	<0.01 *
Smoking	232/964 (24.06%)	25/147 (17%)	207/817 (25.33%)	0.13
Hypertension	650/964 (67.42%)	69/147 (46.93%)	581/817 (71.11%)	<0.001 *
DM	308/964 (31.95%)	50/147 (34.01%)	258/817 (31.57%)	0.27
BMI (kg/m^2^)	30.95 ± 5.31	29.782 ± 5.07	31.164 ± 5.33	
LVEF (%)	45.96 ± 9.45	36.73 ± 10.75	47.82 ± 8.17	<0.001 *
Creatinine (mg/dL)	1.09 ± 0.64	1.54 ± 1.03	1.01 ± 0.50	
CRP (mg/L)	3.99 ± 6.70	8.84 ± 11.59	3.23 ± 5.11	
WBC (×10^3^/μL)	10.83 ± 4.59	13.52 ± 6.3	10.32 ± 3.99	0.001 *
NLR	4.57 ± 4.52	8.76 ± 5.79	3.81 ± 3.79	<0.001 *
SII	825.24 ± 983.85	1717 ± 1611.32	664.68 ± 713.11	
Cholesterol (mg/dL)	176.98 ± 47.56	173.41 ± 45.815	177.62 ± 47.87	0.34
GRACE	139.12 ± 41.89	167.446 ± 34.26	113.55 ± 30.10	<0.001 *
Hs-troponin (pg/mL)	1627.92 ± 2849.62	3620.86 ± 5186.601	1269.34 ± 1985.997	
STEMI	380/964 (39.42%)	98/147 (66.66%)	282/817 (34.52%)	
NSTEMI	283/964 (29.36%)	36/147 (24.49%)	247/817 (30.23%)	0.16
UA	301/964 (31.22%)	13/147 (8.84%)	288/817 (35.25%)	<0.001 *
PCI	509/964 (52.80%)	60/147 (40.82)	449/817 (54.96%)	0.001 *
One vessel	211/509 (41.45%)	21/55 (38.18%)	190/454 (41.85%)	0.66
Two vessels	153/509 (30.5%)	17/55 (30.9%)	136/454 (29.95%)	0.75
Three vessels	145/509 (28.48%)	22/55(40%)	123/454 (27.09%)	0.04 *
No PCI	455/964 (47.19%)	92/147 (62.58%)	363/817 (44.43%)	0.002 *
No CAG	239/455 (52.52%)	54/92 (58.69%)	185/363 (50.96%)	0.23
Not amenable to PCI	93/455 (20.44%)	37/92 (40.21%)	56/363 (15.42%)	<0.001 *
No significant lesions	123/455 (27.03%)	1/92 (1.08%)	122/363 (33.60%)	

M, male; DM, diabetes mellitus; BMI, body mass index; LVEF, left ventricular ejection fraction; CRP, C reactive protein; WBC, white blood cells; NLR, neutrophil to lymphocyte ratio; SII, systemic immune-inflammation index; GRACE, Global Registry of Acute Coronary Events 2 Score; Hs-troponin, high sensitivity troponin I; STEMI, ST elevation acute myocardial infarction; NSTEMI, non-ST elevation myocardial infarction; UA, unstable angina; PCI, percutaneous coronary intervention; CAG, coronary angiography; * *p* < 0.05, statistically significant.

**Table 4:** Demographic, clinical, and paraclinical parameters in STEMI patients with and without MACCE.

Parameters	Total (n = 380)	MACCE (n = 98)	Non-MACCE (n = 282)	*p*
Age (years)	64.49 ± 12.84	70.64 ± 12.42	62.35 ± 12.31	<0.001 *
Sex (M)	248/380 (65.3%)	45/98	203/282	
Smoking	104/380 (27.36%)	18/98	86/282	0.02 *
Hypertension	210/380 (55.26%)	39/98 (39.79%)	171/282 (60.63%)	<0.001 *
DM	115 (30.26%)	38/98 (38.77%)	77/282 (27.30%)	0.04 *
BMI (kg/m^2^)	29.87 ± 4.63	29.265 ± 4.60	30.082 ± 4.63	0.134
LVEF (%)	43.72% ± 10.25	34.56 ± 10.50	46.91 ± 8.01	<0.001 *
Creatinine (mg/dL)	1.11 ± 0.61	1.51 ± 0.97	0.97 ± 0.31	
CRP (mg/L)	6.05 ± 8.28	12.63 ± 13.23	4.72 ± 6.10	0.001 *
WBC (×10^3^/μL)	12.68 ± 11.95	14.08 ± 6.19	12.22 ± 4.42	0.01 *
NLR	6.20 ± 5.25	9.31 ± 5.87	5.12 ± 4.56	<0.001 *
SII	1059.02 ± 1171.16	2042.16 ± 1630.77	717.37 ± 691.17	
Cholesterol (mg/dL)	178.52 ± 41.92	177.33 ± 44.97	178.94 ± 40.89	0.74
GRACE 2	165.21 ± 35.22	170.38 ± 34.47	133.66 ± 21.04	0.003 *
hs-Tni (pg/mL)	2700.12 ± 3714.24	4601.34 ± 5923.52	2039.41 ± 2189.87	<0.001 *
PCI	275 (72.36%)	43 (43.87%)	232 (82.26%)	<0.001
One-vessel CAD	126/275 (45.81%)	19/43 (44.18%)	107/232 (46.12%)	0.8
Two-vessel CAD	83/275 (30.18%)	8/43(18.60%)	75/232 (32.33%)	0.06
Three-vessel CAD	66/275 (24%)	16/43 (37.20%)	50/232 (21.55%)	0.07
No PCI	105 (27.63%)	55 (56.12%)	50 (17.73%)	<0.001
No CAG	32 (30.47%)	29 (52.82%)	3 (6%)	
Not amenable to PCI	31 (29.52%)	26 (47.37%)	5 (10%)	<0.001
No significant lesions	42 (40%)	0	42 (84%)	<0.001

M, male; DM, diabetes mellitus; BMI, body mass index; LVEF, left ventricular ejection fraction; CRP, C reactive protein; WBC, white blood cells; NLR, neutrophil to lymphocyte ratio; SII, systemic immune-inflammation index; GRACE 2, Global Registry of Acute Coronary Events (GRACE) 2 Score; hs-Tni, high sensitivity troponin I; ST elevation acute myocardial infarction; major acute cardiac and cerebral events; PCI, percutaneous coronary intervention; CAD, coronary artery disease; CAG, coronary angiography; * *p* < 0.05, statistically significant.

**Table 5:** SII values at different time points in patients with STEMI or with STEMI-PPCI.

Time (h)	SII Mean	Std. Error	95% CI Lower Bound	Upper Bound
With STEMI				
Baseline	1416.13	68.15	1282.022	1550.25
24	1868.02	79.47	1711.63	2024.42
48	995.485	65.23	867.11	1123.86
With STEMI-PPCI				
Baseline	1437.03	86.30	1266.85	1607.22
24	1808.04	91.26	1628.08	1987.99
48	1126.61	73.82	981.05	1272.19

STEMI, ST-elevation myocardial infarction; PPCI, primary percutaneous coronary intervention; SII, systemic immune-inflammation index.

**Table 6:** STEMI and STEMI PPCI Pairwise comparison of SII at three time points (*p* < 0.001).

Time (I)	Time (J)	Mean Difference (I-J)	St. Error	95% CI Lower Bound	95% CI Upper Bound
STEMI					
Baseline	24 h	−451.889 *	54.995	−584.295	−319.483
	48 h	420.649 *	66.325	260.964	580.334
24 h	baseline	451.889 *	54.995	319.483	584.295
	48 h	872.538 *	67.935	708.977	1036.099
48 h	baseline	−420.649 *	66.325	−580.334	−260.964
	24 h	−872.538 *	67.935	−1036.099	−708.977
STEMI PPCI					
Baseline	24 h	−370.999 *	51.900	−496.307	−245.692
	48 h	310.420 *	56.862	173.134	447.706
24 h	baseline	370.999 *	51.900	245.692	496.307
	48 h	681.419 *	56.808	544.262	818.576
48 h	baseline	−310.420 *	56.862	−447.706	−173.134
	24 h	−681.419 *	56.808	−818.576	−544.262

STEMI, ST-elevation myocardial infarction; PPCI, primary percutaneous coronary intervention; systemic immune-inflammation index. * indicates statistically significant difference at *p* < 0.05.

**Table 7:** MACCE–Time interaction in STEMI and in STEMI PPCI patients.

MACCE	Time (h)	SII Mean	St. Error	95% CI Lower Bound	95% Upper Bound
STEMI					
No	Baseline	720.881	81.913	559.677	882.084
	24	1031.277	95.525	843.287	1219.268
	48	515.257	78.407	360.952	669.561
Yes	Baseline	2111.387	108.932	1897.010	2325.764
	24	2704.769	127.034	2454.768	2954.770
	48	1475.713	104.270	1270.511	1680.915
STEMI PPCI					
No	Baseline	730.476	80.756	571.228	889.723
	24	1052.369	85.394	883.976	1220.763
	48	523.554	69.076	387.340	659.769
Yes	Baseline	2143.599	152.545	1842.787	2444.412
	24	2563.705	161.307	2245.615	2881.794
	48	1729.682	130.482	1472.378	1986.986

STEMI, ST-elevation myocardial infarction; PPCI, primary percutaneous coronary intervention; SII, systemic immune-inflammation index; MACCE, major adverse cardiac and cerebrovascular events.

**Table 8:** AUC comparison for SII at three time points as a predictor of MACCE in STEMI.

Parameter	AUC	95% Lower Bound	95% Upper Bound	Sensitivity %	Specificity %	Cutoff (Youden Index)	*p*
SII baseline	0.874	0.836	0.906	88.78	80.14	>866	<0.001
SII 24 h	0.866	0.821	0.903	86.73	81.15	>1022	
SII 48 h	0.787	0.742	0.827	81.44	67.73	>542	

STEMI, ST-elevation myocardial infarction; SII, systemic immune-inflammation index; major adverse cardiac and cerebrovascular events; AUC, area under curve.

## Data Availability

More data are available upon request from the first author.

## Abbreviations

The following abbreviations are used in this manuscript:

ACS	Acute Coronary Syndromes
ANOVA	Analysis of Variance
ARB	Angiotensin Receptor Blocker
AUC	Area Under the Curve
BMI	Body Mass Index
CAD	Coronary Artery Disease
CBC	Complete Blood Count
CCS	Chronic Coronary Syndromes
CI	Confidence Interval
CKD	Chronic Kidney Disease
CRP	C-Reactive Protein
CT	Computed Tomography
CVD	Cardiovascular Disease
ECG	Electrocardiogram
ESC	European Society of Cardiology
GRACE	Global Registry of Acute Coronary Events
Hs-Tni	High-Sensitivity Troponin i
LVEF	Left Ventricular Ejection Fraction
MACCE	Major Adverse Cardiac and Cerebrovascular Events
MACE	Major Adverse Cardiac Events
MI	Myocardial Infarction
MLR	Monocyte to Lymphocyte Ratio
MRI	Magnetic Resonance Imaging
NLR	Neutrophil to Lymphocyte Ratio
NSTEMI	Non-ST-Elevation Myocardial Infarction
OR	Odds Ratio
PCI	Percutaneous Coronary Intervention
PPCI	Primary Percutaneous Coronary Intervention
PLR	Platelet to Lymphocyte Ratio
SD	Standard Deviation
SII	Systemic Immune-Inflammation Index
SPSS	Statistical Package for the Social Sciences
STEMI	ST-Elevation Myocardial Infarction
UA	Unstable Angina
WBC	White Blood Cells
